# The p53 binding protein PDCD5 is not rate-limiting in DNA damage induced cell death

**DOI:** 10.1038/srep11268

**Published:** 2015-06-11

**Authors:** Florian J. Bock, Maria C. Tanzer, Manuel D. Haschka, Gerhard Krumschnabel, Bénédicte Sohm, Katrin Goetsch, Reinhard Kofler, Andreas Villunger

**Affiliations:** 1 Division of Developmental Immunology, Biocenter, Innsbruck Medical University, Innsbruck, Austria; 2 Division of Molecular Pathophysiology, Biocenter, Innsbruck Medical University, Innsbruck, Austria

## Abstract

The tumour suppressor p53 is an important mediator of cell cycle arrest and apoptosis in response to DNA damage, acting mainly by transcriptional regulation of specific target genes. The exact details how p53 modulates this decision on a molecular basis is still incompletely understood. One mechanism of regulation is acetylation of p53 on lysine K120 by the histone-acetyltransferase Tip60, resulting in preferential transcription of proapoptotic target genes. PDCD5, a protein with reported pro-apoptotic function, has recently been identified as regulator of Tip60-dependent p53-acetylation. In an effort to clarify the role of PDCD5 upon DNA damage, we generated cell lines in which PDCD5 expression was conditionally ablated by shRNAs and investigated their response to genotoxic stress. Surprisingly, we failed to note a rate-limiting role of PDCD5 in the DNA damage response. PDCD5 was dispensable for DNA damage induced apoptosis and cell cycle arrest and we observed no significant changes in p53 target gene transcription. While we were able to confirm interaction of PDCD5 with p53, we failed to do so for Tip60. Altogether, our results suggest a role of PDCD5 in the regulation of p53 function but unrelated to cell cycle arrest or apoptosis, at least in the cell types investigated.

Cells are constantly exposed to noxic insults and therefore have evolved complex mechanisms to respond to these challenges to secure survival and genomic integrity. One of these insults is genotoxic stress, which can lead to persisting mutations thereby contributing to the development of cancer^1^. One of the key pathways preventing the propagation of such mutations is engaged by the tumour suppressor p53. Upon stabilisation and activation in response to DNA damage, p53 translocates to the nucleus and induces the transcription of specific target genes as a central component of the DNA-damage response (DDR)^2^. Some of these genes promote arrest of the cell cycle while others assist in the repair of DNA-damage. However, if damaged too severe, genes promoting the induction of apoptosis can eliminate these damaged cells. Together, these mechanisms prevent the propagation of mutations and other transforming events, as evidenced by the increased rate of spontaneous tumours observed in p53-deficient mice^3^, the increased frequency of tumourigenesis in Li-Fraumeni patients (harbouring germline mutations of p53)^4^ and the high frequency of p53 loss-of-function in human cancer^5^. However, in recent years, more and more p53 target genes have emerged which cannot be classified as either direct regulators of cell cycle arrest or cell death-inducers, but rather represent modulators of diverse processes like metabolism, metastasis or autophagy^6^. Furthermore, accumulating evidence suggests that the tumour suppressor function of p53 is restricted to a subset of target genes that do not seem to control cell cycle arrest or apoptosis directly, contrasting general belief^7^,^8^,^9^,^10^ and re-igniting the search for relevant p53-target genes and response modifiers.

After DNA damage activates the DDR machinery, p53 is phosphorylated at several amino acids^11^,^12^. These posttranslational modifications prevent its recognition and subsequent degradation by the E3 ubiquitin ligase MDM2 and enable p53 to translocate to the nucleus and induce transcription of its target genes. However, it is still rather unclear how p53 actually “senses” the level of damage and selectively controls gene expression. Acetylation of p53 on lysine (K) 120 has been shown to enable transcription of proapoptotic target genes like Puma or Bax^13^,^14^. This modification is carried out by the histone acetyltransferase (HAT) Tip60, which is activated in response to DNA damage by methylated histone H3^15^.

One of the modulators of the HAT activity of Tip60 described is programmed cell death 5 (PDCD5)^16^, a protein previously reported to be induced in response to and implicated in the execution of DNA damage triggered apoptosis^17^,^18^,^19^,^20^,^21^,^22^. According to literature, PDCD5 is transcriptionally upregulated in response to genotoxic stress and translocates to the nucleus where it is part of a complex containing Tip60 and p53^16^,^23^, thereby enhancing the HAT activity of Tip60. This in turn is thought to lead to increased K120 acetylation and increased transcription of proapoptotic genes promoting cell death. In addition to this reported nuclear control of the transcriptional response of p53, PDCD5 also prevents ubiquitination of p53 in the cytoplasm by sequestering MDM2, thereby increasing p53 stability^23^. Given this dual role, it is interesting to note that the expression levels of PDCD5 are deregulated in several cancers, including ovarian carcinoma, chondrosarcoma, prostate cancer, leukemia and glioma^24^,^25^,^26^,^27^,^28^. In line with a putative tumour suppressive role of PDCD5, adenoviral overexpression in leukemic cell lines prevented their outgrowth in mouse xenotransplantation models^29^. Mechanistically, PDCD5 appears to perturb mitochondrial integrity by promoting Bax activation and caspase-3 activity that reportedly is reduced in cells where PDCD5 was knocked down by RNA interference prior to exposure to DNA damage^17^. Although these findings are in line with a modulatory role on p53 activity, structural analysis and studies using the recombinant proteins suggested that the N-terminal domain of PDCD5 can exert a pro-death role on its own that may be independent from its proposed modulatory activity of p53^30^.

To explore the role of PDCD5 in cell death signalling in more detail we generated a PDCD5-specific antiserum that recognizes endogenous mouse and human PDCD5 protein as well as a series of human cancer cell lines where PDCD5 expression can be conditionally ablated by RNAi and studied their response to DNA damage.

## Results

### PDCD5 is not a transcriptional target of the DDR

**Figure 1:**
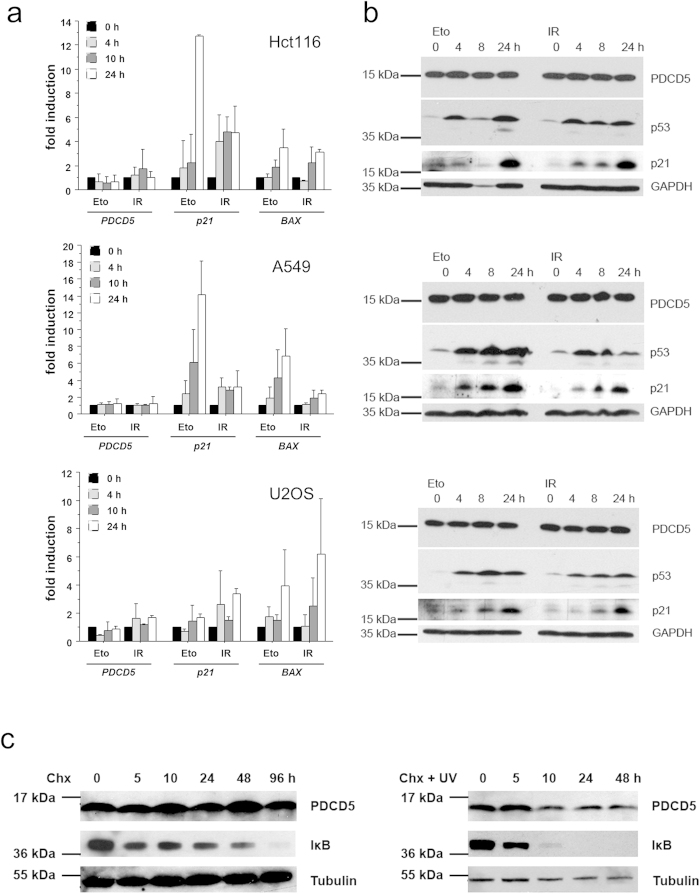
Analysis of PDCD5 expression in response to DNA damage. (**a**) Hct116, A549 and U2OS cells were treated with 10 μg/ml etoposide or irradiated with 10 Gy. mRNA levels of *PDCD5, p21* and *BAX* were determined by RT-qPCR. Bars represent means of n = 2 experiments performed in duplicates ± SD. (**b**) Hct116, A549 and U2OS cells were treated as indicated and protein lysates prepared at the indicated times for immunoblotting. (**c**) Hct116 cells were treated with 20 μg/ml cycloheximide or cycloheximide plus 10 mJ UV irradiation and protein lysates were prepared. IκB degradation was monitored as a control for successful treatment. Western blots shown are representatives of two independent experiments yielding comparable results.

In an effort to study the role of PDCD5 in the DNA damage response, we first investigated the transcriptional regulation of PDCD5 in Hct116 colon carcinoma, A549 lung carcinoma and U2OS osteosarcoma cells. Cells were exposed to γ-irradiation or etoposide, two established triggers of p53 and the DDR machinery. However, neither treatment led to a consistent and significant increase in PDCD5 mRNA transcripts while the p53 target genes p21 and Bax where found increased (Fig. 1a, Suppl. Figure 1a). To confirm these findings, we also determined PDCD5 protein levels. In accordance with our transcriptional analysis we did not detect any significant or reproducible differences in PDCD5 protein after DNA damage (Fig. 1b, Suppl. Figure 1b). In contrast, p53 was stabilized and p21 induced, showing induction of the DDR pathway. Furthermore, when we investigated protein stability of PDCD5 in the absence or presence of UV-induced DNA damage and cycloheximide (CHX) treatment, we found it to have a very long half-life. In contrast, IκB, a component of the NF-κB signalling cascade, was rapidly degraded under these conditions (Fig. 1c). Altogether, these results provide evidence that PDCD5 is not significantly regulated upon genotoxic stress at the transcriptional or post-translational level, at least not in the cell lines analyzed.

**Figure 2:**
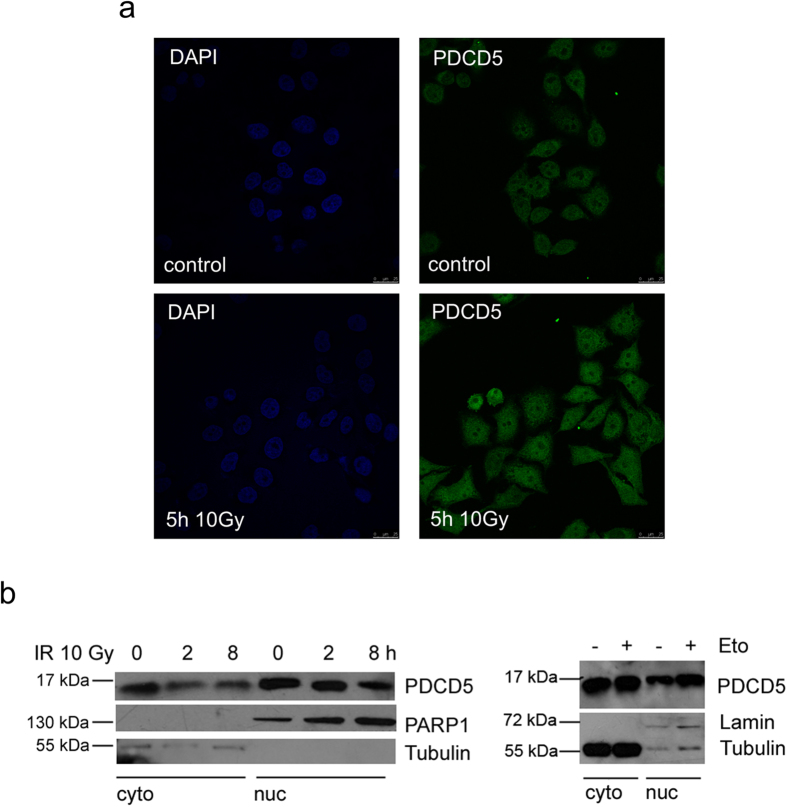
Localisation of PDCD5 in response to DNA damage. (**a**) HeLa cells were γ-irradiated and immunostaining was performed with antibodies recognising PDCD5. DAPI was used to stain the nucleus. One of two experiments yielding similar results shown. (**b**) 293T cells were separated into cytoplasmic and nuclear fractions after treatment with γ-irradiation or etoposide for the indicated times. PDCD5 levels were determined by immunoblotting, where tubulin and PARP1 or Lamin served as loading and quality control of the subcellular fractions generated. Western blots shown are representatives of two independent experiments yielding comparable results.

As PDCD5 was reported to be detectable in the cytoplasm as well as in the nucleus and described to shuttle into the nucleus upon DNA damage^18^, we also evaluated subcellular localization of PDCD5. Indeed, we were able to detect PDCD5 in the cytosol and in the nucleus by immunofluorescence staining using two different antibodies recognising PDCD5 (Fig. 2a, data not shown). In addition, upon irradiation PDCD5 staining intensity appeared mildly increased in the nuclei of cells. However, using biochemical fractionation and immunoblotting, we could only appreciate a decrease in cytoplasmic PDCD5 but no significant increase in the nuclear fraction. Of note, etoposide treatment did not cause a significant drop in cytoplasmic PDCD5 levels nor did it trigger a pronounced increase in the nuclear fraction (Fig. 2b). Together, this demonstrates that PDCD5 is available in the cytoplasm and nucleus in steady state, but does not show a clear preference after DNA damage.

### PDCD5 interacts with p53 but not Tip60

**Figure 3:**
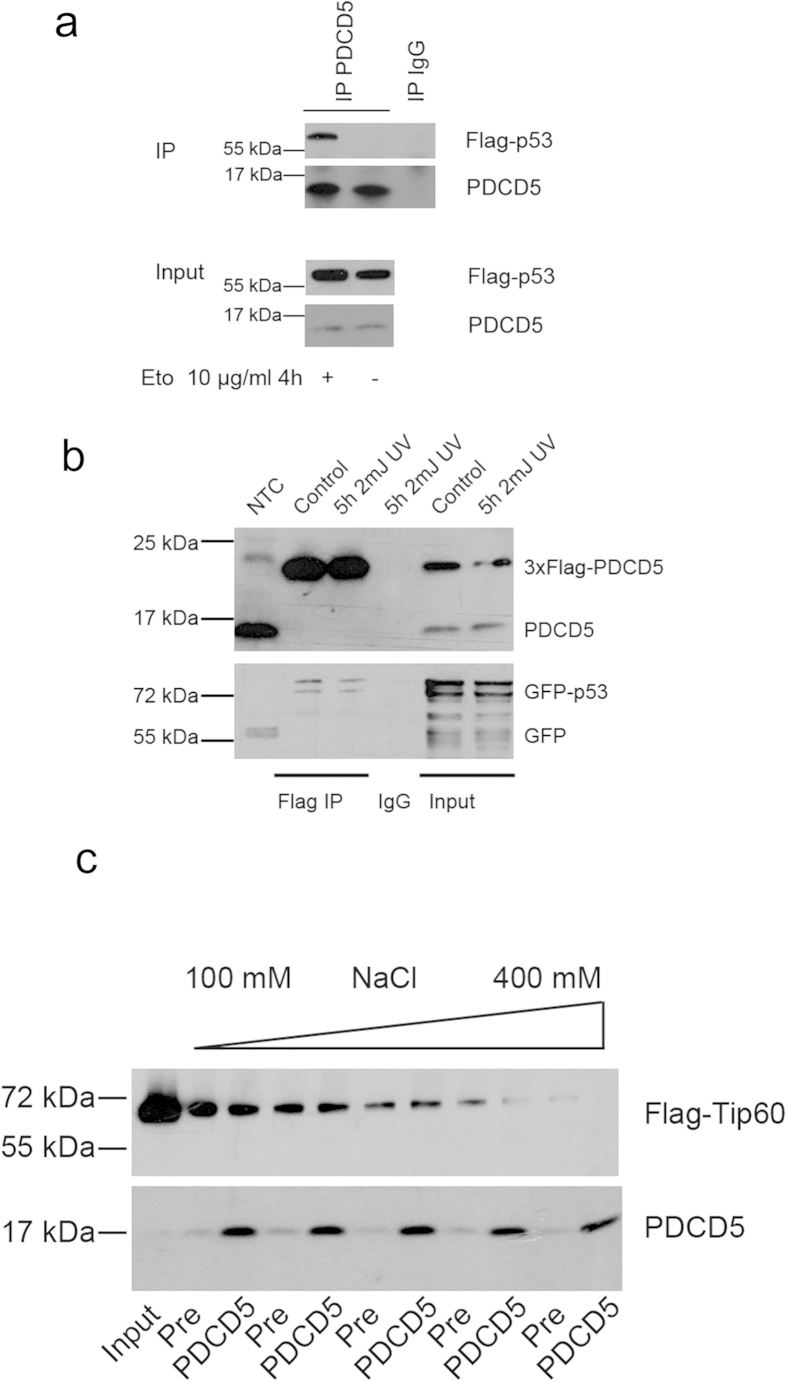
PDCD5 interacts with p53, but not Tip60. (**a**) 293T cells were transfected with Flag-p53, left untreated or exposed to etoposide for 4 h and immunoprecipitation was performed using an anti-PDCD5 antiserum. One of two experiments yielding similar results is shown. (**b**) 293T cells were transfected with 3xFlag-PDCD5 and GFP-p53, left untreated or were exposed to UV for 4 hours. Immunoprecipitation was performed using anti-Flag-beads. One of two experiments yielding similar results is shown. (**c**) 293T cells were transfected with Flag-Tip60 and immunoprecipitated with PDCD5 antiserum or pre-immuneserum as control. After immunoprecipitation the beads were distributed equally into different tubes, washed with buffers differing only in their salt concentration and immunoblotted.

As PDCD5 was described to bind to both p53 and Tip60, we used co-immunoprecipitation assays to re-investigate these postulated interactions. In accordance with previous findings^16^,^23^, we could detect interaction of endogenous PDCD5 with Flag-tagged p53 in response to etoposide treatment in 293T cells (Fig. 3a). Furthermore, PDCD5 bound to p53 upon overexpression of both proteins already in untreated cells as well as after UV-irradiation (Fig. 3b).

In contrast, we were not able to reproducibly detect specific interaction between PDCD5 and Tip60 using several immunoprecipitation-protocols and different stimuli tested (data not shown). In fact, Tip60 was easily precipitated using pre-immune serum conjugated to sepharose-beads. Using different concentrations of salt during washing, we found a dose dependent decrease of this interaction while PDCD5/Ig complexes where highly stable, even at 400 mM of salt in the wash buffer (Fig. 3c). Although not excluding interaction between PDCD5 and Tip60 completely, this experiment shows that the interaction of PDCD5 and TIP60 is dependent on very specific experimental conditions. Of note, these experiments were conducted under conditions previously described to enable detection of Tip60 and PDCD5 interaction^16^.

### Normal Transcription and DNA damage response in PDCD5 knockdown cells

**Figure 4:**
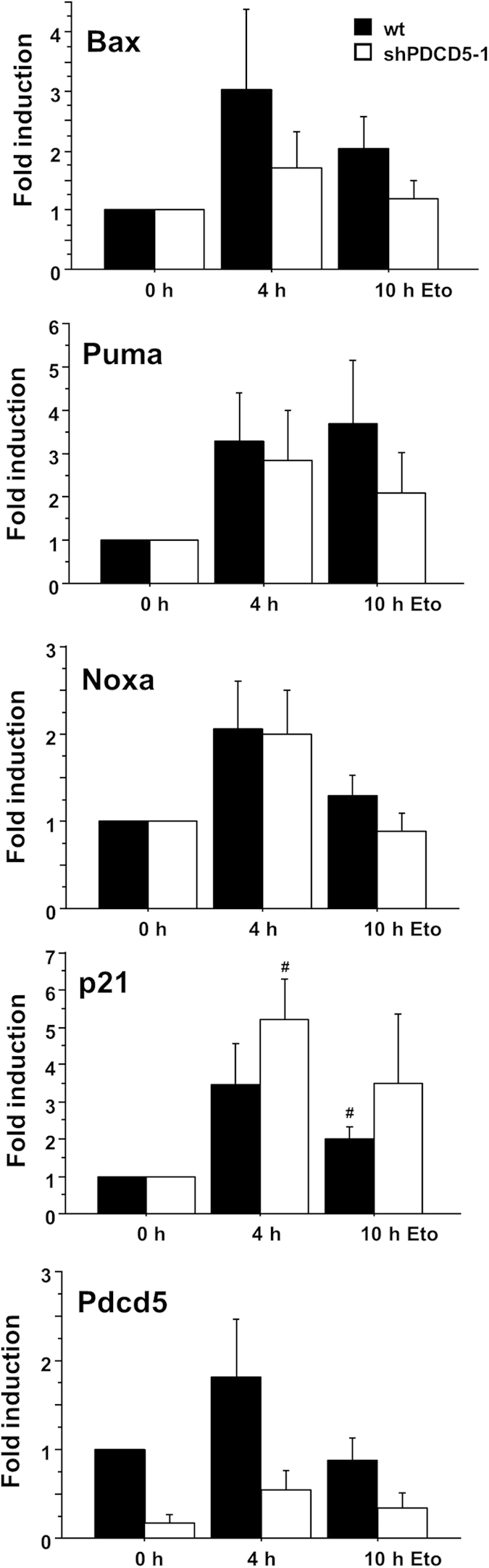
PDCD5 does not influence transcription of p53 target genes. Hct116 cells were treated with etoposide for various times and qPCR was performed for the indicated target genes on cDNA prepared from RNA. Fold induction compared to untreated controls was determined with the ^ΔΔ^Ct method using actin as normalising gene. Bars represent mean ± SEM; n ≥ 3; # p < 0.05 vs untreated.

We next investigated whether lack of PDCD5 influences transcription of p53 target genes involved in the DNA damage response. Hct116 cells with an inducible knockdown of PDCD5 were treated with Doxycycline for 5 days to deplete PDCD5 and were then exposed to etoposide. Expression of known p53 target genes was analysed by quantitative RT-PCR. Proapoptotic *Bax*, *Noxa* and *Puma* as well as the CDK inhibitor *p21* were upregulated to various extents in response to DNA damage and in a time dependent manner. However, no significant effect of PDCD5 knockdown was detected using cell lines harbouring one of two independent shRNAs targeting PDCD5 (Fig. 4, data not shown).

**Figure 5:**
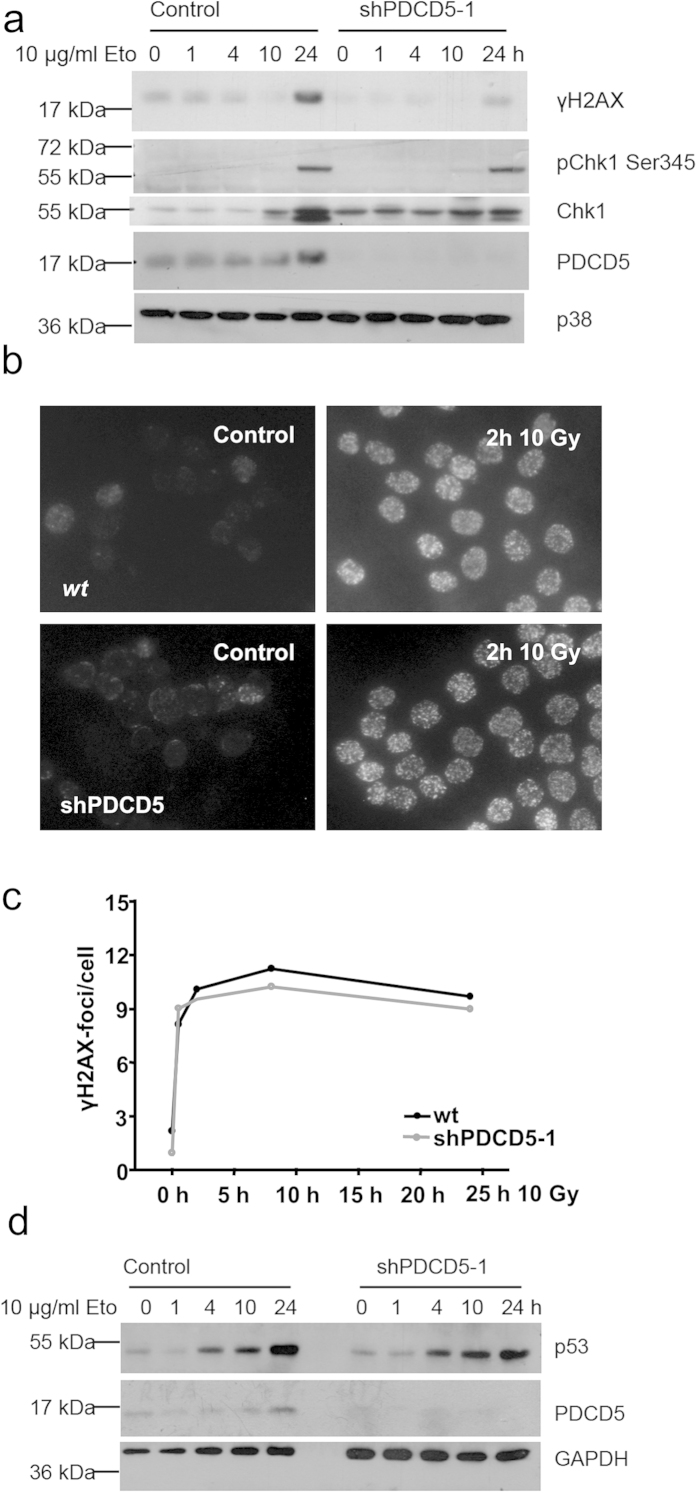
Normal DNA damage response in PDCD5 deficient cells. (**a**) Hct116 cells carrying an inducible PDCD5 shRNA were treated with etoposide for the indicated times after PDCD5 knockdown. Lysates were prepared and immunoblotted for the indicated proteins. One of two experiments yielding similar results is shown. (**b**) HeLa cells grown on cover slips were irradiated, fixed after 2 h and stained for γH2A.X. (**c**) Quantification of data from (**b**) as determined by Cell Profiler analysis. Data represent mean of >200 cells analysed in two independent experiments. (**d**) Hct116 cells with an inducible shRNA targeting PDCD5 were treated with etoposide for the indicated times, protein lysates were prepared and immunoblotted for the indicated proteins. One of two experiments yielding similar results is shown.

The putative PDCD5 interaction partner Tip60 was not only described to bind and acetylate p53, but also to be essential for acetylation and activation of the DNA damage response kinase ATM after genotoxic stress^31^. We therefore evaluated the impact of PDCD5 depletion on downstream targets of ATM. One of the key proteins phosphorylated by ATM is the histone variant H2A.X. Immunoblotting for the phosphorylated form, γH2A.X, showed decreased levels in PDCD5 knockdown cells. Furthermore, phosphorylation of the DNA damage response kinase Chk1 appeared unaffected (Fig. 5a), although total levels of Chk1 increased more in control cells compared to PDCD5 knockdown cells. To clarify this discrepancy, we also investigated the accumulation of γH2A.X foci in response to irradiation by immunofluorescence. In contrast to decreased levels of γH2A.X detected by immunoblotting, we were unable to see any differences in the number and kinetics of foci formation in PDCD5 knockdown cells (Fig. 5c,d). However, differences in foci size that would explain the difference noted in immunoblotting were not appreciable in our analysis. Of note, stabilisation of p53 was also intact in response to etoposide treatment upon PDCD5 knockdown in Hct116 cells (Fig. 5d).

### PDCD5 is dispensable for cell death upon genotoxic stress

**Figure 6:**
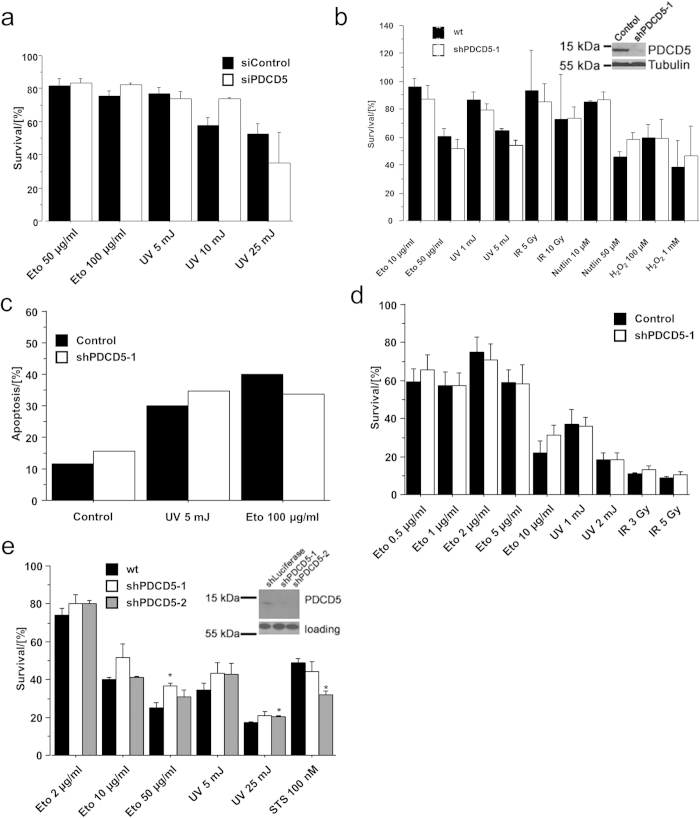
Normal cell death in response to DNA damage in PDCD5 knockdown cells. (**a**) 293T cells were transfected with siRNA targeting PDCD5 and treated as indicated. 24 h after treatment, MTT assay was performed and metabolic activity determined relative to untreated controls. Data represent mean ± SEM; n = 3. (**b**) Hct116 lines harbouring an inducible shRNA targeting PDCD5 were treated as indicated. After 24 h MTT assay was performed and survival determined relative to untreated cells. Data represent mean ± SEM; n = 3–6. (**c**) The same Hct116 lines as in (**b**) were treated as indicated and apoptosis was determined by AnnexinV/PI staining after 24 h. Data represent mean; n = 2–6. (**d**) The same Hct116 lines as in (**b**) were treated as indicated and colony formation was determined as specified in the material and methods section. Survival was calculated relative to untreated controls. Data represent mean ± SEM; n ≥ 3 (**e**) SV40 immortalised wt MEF harbouring two different constitutive shRNAs targeting PDCD5 were treated as indicated. 24 h after treatment, MTT assay was performed and survival determined relative to untreated cells. Data represent mean ± SEM; n = 3; ^*^ p < 0.05 vs wt.

We next investigated the cellular response upon genotoxic stress in cells with knockdown of PDCD5. In a first attempt, we used siRNA to knock down PDCD5 in 293T cells and determined metabolic activity, serving as an indirect measure of viability, in response to DNA damage by MTT assay. Cells transfected with scramble or PDCD5-specific siRNA succumbed to cell death in a comparable manner after treatment with various DNA damage-inducers (Fig. 6a). In an independent approach, we generated inducible PDCD5-shRNA Hct116 cell lines using two different shRNA sequences. Both shRNA lines were used to test the impact of PDCD5 knockdown on etoposide, UV or γ-IR treatment, as well as stimulation with Nutlin-3, an inhibitor of MDM2 leading to p53 dependent cell death. In accordance with our previous results, we could not detect any significant differences in metabolic activity in response to the treatments used (Fig. 6b, Suppl. Figure 2a). To determine cell death directly, we measured cells undergoing apoptosis by Annexin-V and propidium iodide staining followed by flow cytometric analysis (Fig. 6c), corroborating our results from the MTT assay.

As minor differences in viability may only become visible in more selective assays such as those monitoring clonal survival, we also conducted colony-forming assays after DNA-damage. Colonies were visualised by crystal violet staining, solubilised and the optical density (OD) of the cell lysate was measured at 595 nm for the assessment of clonogenic potential relative to untreated cells. Again the data showed no difference between wild type and PDCD5 knockdown cells even in this long-term assay in both Hct116 (Fig. 6d, Suppl. Figure 2b) and U2OS cells (Suppl. Figure 2c). To extend our findings to other species, we repeated MTT assays in SV40-immortalised wild type mouse embryonic fibroblasts (MEF) with stable knockdown of PDCD5 using two distinct shRNAs. Different DNA damaging treatments showed no general influence of PDCD5 knockdown on cellular fitness and metabolic activity (Fig. 6e), providing additional evidence that PDCD5 is dispensable for this process in mouse and human cells.

**Figure 7:**
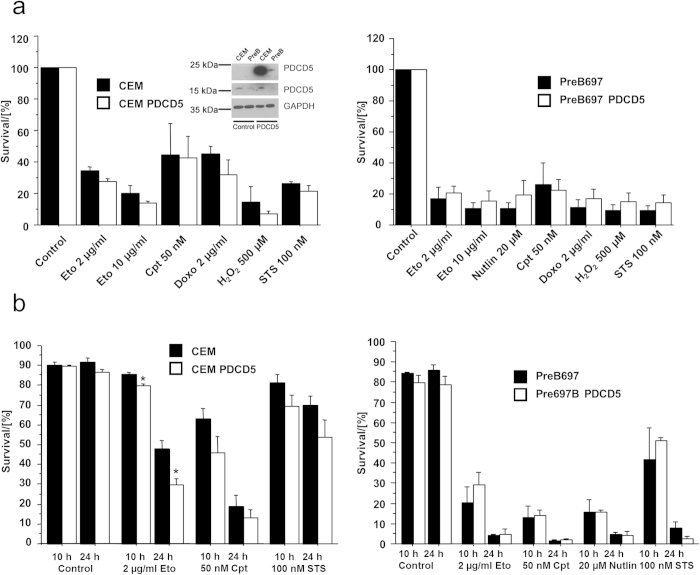
Cell death in response to DNA damage and PDCD5 overexpression. (**a**) CEM and PreB697 lymphoma cell lines overexpressing 3xFlag-PDCD5 were treated as indicated. 24 h after treatment, MTT assay was performed and survival determined relative to untreated cells. Data represent mean ± SEM; n = 3. (**b**) The same cells as in (**a**) were treated as indicated and after 10 h and 24 h cell death was determined by AnnexinV/7AAD staining. Data represent mean ± SEM; n = 3; ^*^ p < 0.05 vs wt.

Since our approach so far was to investigate the loss of function of PDCD5 in cell death, we finally generated two acute lymphoblastic leukemia (ALL) cell lines (PreB697 and CEM) stably overexpressing 3xFlag-PDCD5. Overexpression in CEM cells was much more pronounced when compared to PreB697 cells but did not seem to affect cell growth. Despite increased levels of PDCD5 in these cell lines, there was no difference in viability as measured by MTT assay (Fig. 7a) or AnnexinV/7-AAD staining (Fig. 7b) upon treatment with different DNA damaging regimens, however CEM cells, that showed very strong overexpression, were slightly more sensitive towards etoposide, but responded normal to camptothecin. We conclude that PDCD5 is not rate limiting for cell death induction upon DNA damage, but may sensitize to apoptosis when highly overexpressed.

### PDCD5 knockdown does not affect the cell cycle in response to DNA damage

**Figure 8:**
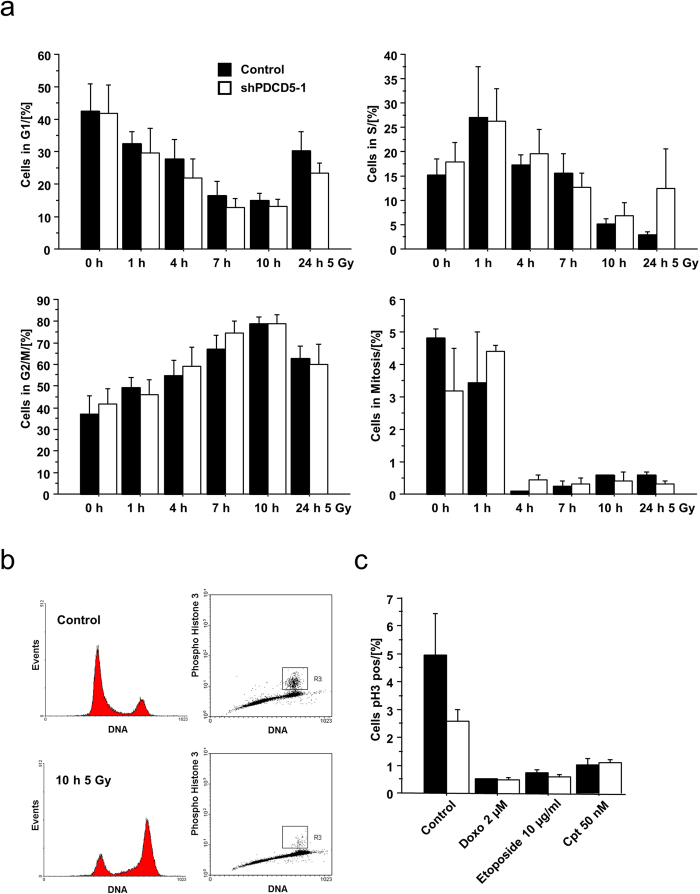
Knockdown of PDCD5 does not alter cell cycle distribution. (**a**) Hct116 cells with inducible PDCD5 shRNA were treated with doxycycline for 5 days followed by γ-irradiation for the indicated times. Cell cycle distribution was assessed by combined PI and α-Histone H3 (Ser10) staining and quantified by flow cytometry. Data represent mean ± SEM; n = 3-4. (**b**) Representative FACS profiles of Hct116 cells left untreated or exposed to 5 Gy of irradiation and analysed 10 h later. (**c**) Hct116 cells with inducible PDCD5 shRNA were treated with the indicated DNA damaging agents and cell cycle distribution was determined by combined PI and pH3 staining and FACS analysis. Data represent mean ± SEM, n = 3.

Clearly p53 not only regulates cell death but also enforces cell cycle arrest. We therefore investigated the effect of PDCD5 knockdown on cell cycle distribution after DNA damage. Time-course analysis in response to irradiation showed a similar cell cycle distribution in Hct116 cells, as assessed by DNA staining, as well as cells in mitosis, quantified by intracellular staining of phospho-Histone H3 (Fig. 8a, Suppl. Figure 3). To rule out the possibility that PDCD5 is required for cell cycle arrest only in response to certain genotoxic agents, we looked at pH3 staining also in response to etoposide, doxorubicin or camptothecin treatment (Fig. 8b). All of the treatments resulted in cell cycle arrest independent of the presence or absence of PDCD5 in Hct116 cells.

## Discussion

The transcription factor p53 is considered to execute its tumour suppressing function via the modulation of two opposing but interwoven signalling pathways, i.e. cell cycle arrest and apoptosis^5^. Although the knowledge about the role of p53 in these processes has made substantial progress in the last decades, several details are still rather poorly understood. One of these issues is how p53 decides which response should be triggered upon DNA-damage and how this can be achieved. Evidence accumulates that these decisions are modulated by posttranslational modifications. Acetylation of K120 on p53 by Tip60 increases binding of p53 to proapoptotic target gene promoters, thereby driving cells preferentially into apoptosis^13^,^14^. In the current model, DNA damage induces phosphorylation of Heterochromatin protein 1 beta (HP1β) by Casein kinase 2 (CK2)^32^, thereby releasing HP1β usually bound to methylated Histone H3 on chromatin. Subsequently Tip60 binds to the now free methylated Histone H3 and becomes activated^15^, acetylatying ATM^31^ and thereby assists in its activation. Hence, the portion of active Tip60 may correlate directly with the number of sustained DNA lesions and it may act as a sensor “quantifying” the severity of the damage obtained to downstream effectors in a proportional or amplifying manner.

PDCD5 was proposed to be an additional intermediate fine-tuning this pathway by increasing the HAT activity of Tip60 after binding, thereby promoting K120 acetylation of p53 and promoting cell death in response to DNA damage^16^. However, our studies suggest that i) PDCD5 mRNA and protein are not controlled by the DDR directly (Fig. 1), ii) it does not preferentially accumulate in the nucleus under these conditions (Fig. 2) and iii) that the reported protein-protein interactions rely on particular experimental conditions, as we noted strong binding of Tip60 to sepharose beads (Fig. 3c), questioning specificity of reported results^16^. Furthermore, PDCD5 reportedly also binds the promoter of *p21*, but it is not clear if it is recruited to this promoter by p53 directly^23^. Clearly, PDCD5 can bind to p53 in response to DNA damage (Fig. 3), thereby possibly preventing MDM2 binding and ameliorating proteasomal degradation of p53, as proposed by Xu *et al.*^23^.

Despite its ability to interact with p53 (Fig. 3a,b), we failed to observe a significant effect of PDCD5 knockdown on the transcription of a number of critical proapoptotic and cell survival genes regulated by p53 after DNA damage (Fig. 4). However, it should be noted that the investigated genes comprise only a small number of described p53 targets^33^ and it is still formally possible that PDCD5 regulates the transcription of other p53 target genes not examined in our analysis. However, we consider this, at least in light of the “classical” role of p53 in inducing DNA damage dependent cell cycle arrest and apoptosis, of limited relevance, as we failed to observe any significant impact on cell cycle arrest or apoptosis at the cellular level using three independent assays with two independent cell lines and shRNA sequences. While the reason for the discrepancies with published results implicating PDCD5 in cell death are currently unclear, but may be due to variations in experimental conditions or cell systems used, they rule out a more general rate-limiting role for PDCD5 in cell cycle arrest and apoptosis upon DNA-damage. As PDCD5 was also described to potentially modulate some aspects of cell death induction via the mitochondrial pathway^17^, it might be required for other, yet unexplored triggers or pathways inducing cell death. For example, p53 was also reported to directly activate Bax at the mitochondria^34^ or contribute to cell death caused by necrosis, triggered by reactive oxygen species^35^. Thus, it remains possible that PDCD5 may act selectively in these responses, but our preliminary analysis argues against a role for PDCD5 in necrosis triggered by excessive ROS production (Figs 6b and 7a). Hence, for now, we need to conclude that PDCD5 does not play a major role in cell cycle arrest or apoptosis in response to DNA damage.

## Methods

### Chemicals, Cytokines and antibodies

Chemicals and cytokines used were staurosporine, cycloheximide, doxorubicin, DAPI, H_2_O_2_, Nutlin-3, polybrene, propidium-iodide, doxocycline, puromycin (all Sigma), AnnexinV–APC, AnnexinV-488, 7-AAD (Biolegend), etoposide, camptothecin (Alexis). Antibodies: α-IκBα (#9242), α-PARP1 (#9532), α-GAPDH (#2118), α-Histone H3 (Ser10) (#9701), α-γH2A.X (#2577), pChk1 Ser345 (#2341), Chk1 (#2345) (Cell Signaling); α-p53 (sc-6243), α-tubulin (sc-32293) (Santa Cruz Biotechnology); α-PDCD5 (Proteintech); α-LaminB1 (gift from Peter Gruber); α-Flag (M2) (Sigma), α-p21 (#554262) (BD Pharmingen); α-rabbit AlexaFluor 488 (Invitrogen), goat α-rabbit HRP, goat α-mouse HRP (Dako). SiRNA targeting human PDCD5 (5′-CUAAAGCAGUAGAGAAUUATT-3′) was synthesised by Microsynth and transfected into 293T cells using Metafectene (Biontex) according to the manufacturer′s instructions.

### Generation of an anti-PDCD5 antiserum

Mouse full-length PDCD5 tagged with GST was purified from *E.coli* with Glutathione-sepharose using standard protocols. Specificity was confirmed by siRNA experiments using 293T cells transiently transfected with Flag-tagged mouse PDCD5 (Suppl. Figure 1b). The antiserum, in contrast to the commercial antibody used, recognizes mouse as well as human PDCD5 (Fig. 6). The immunization strategy for antiserum generation was described previously^36^.

### Immunoblotting

Cells were washed with PBS, harvested by trypsinization, washed again in PBS and lysed in RIPA buffer (150 mM NaCl, 50 mM Tris, 1% v/v NP40, 0.5% v/v sodium deoxycholate, 0.1% v/v SDS) and used for protein quantification with Bradford reagent (Pierce). Usually, 50 μg of the lysates were loaded and run on Tricine gels^37^ and subsequently transferred to Hybond ECL nitrocellulose membranes (GE Healthcare) by electroblotting. After incubation with the indicated antibodies, proteins were visualised by chemoluminescence (Pierce).

### Nuclear Extracts

Nuclear Extracts were prepared as described previously^36^. Briefly, cells were harvested after treatment at the indicated time points and were lysed in a cytosolic lysis buffer containing 10 mM Hepes, pH 7.9, 10 mM KCl, 0.1 mM EDTA, 0.1 mM EGTA, 1 mM DTT, and 0.5 mM PMSF. Nuclei were pelleted by centrifugation, washed and lysed for 30 minutes at 4 °C in a nuclear buffer containing 20 mM Hepes, pH 7.9, 0.4 M NaCl, 1 mM EDTA, 1 mM EGTA, 1 mM DTT and 1 mM PMSF. Nuclear extracts were centrifuged to obtain the solubilized nuclear fraction and both fractions analysed by western blotting.

### Immunoprecipitation

293T cells were transfected with the indicated constructs (Flag-PDCD5, Flag-p53 (Addgene #10838), Flag-Tip60) for 24 h with Metafectene (Biontex) according to the manufacturer′s instructions and subsequently treated as indicated. Cells were lysed in IP-Buffer^16^ (300 mM NaCl, 50 mM Tris pH 8.0, 0.4% NP-40, 10 mM MgCl_2_, 2.5 mM CaCl_2_ and protease inhibitors (Roche)) and immunoprecipitated with Flag-M2 beads (Sigma) or PDCD5 antiserum coupled overnight to Affi-Prep Protein A beads (Biorad). Mouse IgG (Santa Cruz Biotechnology) or pre-immuneserum served as controls, respectively. After incubating the lysate with antibody-coupled beads for 1 h, beads were washed three times in lysis buffer, unless otherwise indicated. Beads were boiled in SDS loading buffer and supernatant loaded on SDS-Page gels.

### Quantitative RT-PCR

RNA was isolated with Trizol (Invitrogen) and after DNAse digestion (Promega) reversely transcribed (Omniscript, Qiagen) using random hexamer primers. cDNA was amplified with DyNAmo Flash SYBR Green qPCR Kit (Finnzymes) on an Eppendorf Mastercycler ep realplex2 cycler (40 cycles with 10 s 94 °C, 30 s 62 °C, 30 s 72 °C). Primers used were hPDCD5 (forward 5′- GCT GCA GGC CAA ACA C -3′; reverse 5′- CTG TTT GTT GGC TTA CTT TTT TAA GG -3′), hBax (forward 5′- TTT TGC TTC AGG GTT TCA TC -3′; reverse 5′- CAG TTG AAG TTG CCG TCA GA -3′), hPuma (forward 5′- TCA ACG CAC AGT ACG AGC G -3′; reverse 5′- TGG GTA AGG GCA GGA GTC C -3′), hNoxa (forward 5′- AGA GCT GGA AGT CGA GTG T -3′; reverse 5′- GCA CCT TCA CAT TCC TCT C -3′), hp21 (forward 5′- TTG TAC CCT TGT GCC TCG CT -3′; reverse 5′- TTG GAG AAG ATC AGC CGG C -3′) and actin (forward 5′- ACT GGG ACG ACA TGG AGA AG -3′; reverse 5′- GGG GTG TTG AAG GTC TCA AA -3′). Fold induction compared to untreated controls was calculated by the ^ΔΔ^CT method.

### Immunofluorescence

Immunofluorescence staining was performed as previously described^38^: HeLa cells were seeded in 6-wells onto sterilized glass cover slips and left to adhere overnight. Then cells were treated as indicated and after various time points washed with PBS and fixed with 4% PFA in PBS for 20 min at RT. Following three washes with PBS, cells were concurrently permeabilised and unspecific binding blocked by 1 h incubation with PBS containing 0.1% Tween20, 1% BSA and 10% FCS. Next, cells were incubated with α-PDCD5 or γH2A.X antibody (1:100) in blocking solution at 4 °C overnight, washed again for three times with PBS and incubated with α-rabbit AlexaFluor 488 secondary antibody in blocking solution for one hour at room temperature. Finally, cells were briefly washed again, incubated with DAPI (2 μg/ml) for 10 min and mounted on microscope slides with Vectashield. Images were acquired on a confocal laser-scanning microscope (Leica SP5) and γH2A.X foci per cell were evaluated using Cell Profiler software^39^.

### Survival assay, cell cycle analysis, colony formation and MTT assay

Cell viability was determined by staining with 7-AAD and AnnexinV-APC or AnnexinV-488 (Biolegend) in AnnexinV-binding buffer (10 mM Hepes pH 7.4, 140 mM NaCl, 2.5 mM CaCl_2_) and analysed on a FACSCalibur instrument (Beckton Dickinson). Cells negative for 7-AAD and AnnexinV were considered as alive. For cell cycle analysis, cells were fixed in 70% EtOH/PBS, blocked in PBS with 1% BSA, permeabilised with 0.25% Triton X-100 in PBS and stained with rabbit α-Histone H3 (Ser10) antibody (1:100), followed by α-rabbit AlexaFluor 488 secondary antibody (1:100). After digestion with RNAseA and staining with 40 μg/ml propidiumiodide (30 min 37 °C), cells were analysed by flow cytometry on a FACScan instrument. For the MTT assay, 5.000 cells (MEF, Hct116) or 10.000 cells (CEM, PreB697) were plated in 96-wells in either medium containing the indicated drugs (Lymphoma lines) or were treated the following day as indicated. MTT assay (Roche) was performed 24 h after treatment according to the manufacturer′s instructions.

For clonogenic survival assays, cells were plated and treated as indicated. After 6 days colonies were visualised by staining with 0.2% crystal violet in 50% methanol and quantified by solubilisation in 1% SDS in PBS and OD-measurement at 595 nm.

### Cell lines

Hct116 and U2OS cell lines with a TetR repressor were kindly provided by Stephan Geley, SV40 MEF were generated by standard methods^36^ and cultured in DMEM (Sigma) supplemented with 10% FCS (PAA), Penicillin/Streptomycin (Sigma) and 250 μM L-Gln (Invitrogen). To generated inducible shPDCD5 knockdown lines, shRNAs (human: shPDCD5-1: 5′- GCA GAA ATG AGA AAC AGT ATC -3′; shPDCD5-2 5′- CAG ATG GCA AGA TAT GGA CAA -3′; mouse: shPDCD5-1: 5′- GCA GAA ATG AGA AAC AGT ATC -3′; shPDCD5-2 5′- GAA CAA GGT TTG ATA GAA A -3′) were cloned into the lentiviral vector pHR-Dest-SFFV-puro^40^ by Gateway cloning (Invitrogen). Stable cell lines were generated by lentiviral transduction of Hct116, U2OS and MEF cells followed by selection with puromycin. To induce PDCD5 knockdown, cells were treated with 1 μg/ml doxocyclin for 5 days before assays were performed. A549 cells were cultured in DMEM (Sigma) supplemented with 10% FCS (PAA), Penicillin/Streptomycin (Sigma) and 250 μM L-Gln (Invitrogen). Lymphoma cell lines PreB697 and CEM were cultured in RPMI 1640 Medium supplemented with 10% FCS (PAA), Penicillin/Streptomycin (Sigma) and 250 μM L-Gln (Invitrogen). PDCD5 overexpressing lines were generated by Lentiviral transduction with 3xFlag-PDCD5^41^.

### Statistical Analysis

For statistical analysis an unpaired t-test was used using StatView software program.

## Additional Information

**How to cite this article**: Bock, F. J. *et al.* The p53 binding protein PDCD5 is not rate-limiting in DNA damage induced cell death. *Sci. Rep.* **5**, 11268; doi: 10.1038/srep11268 (2015).

## Supplementary Material

Supplementary Information
