# Supplementary material for: The p53 binding protein PDCD5 is not rate-limiting in DNA damage induced cell death
Source: Sci Rep. 2015 Jun 11;5:11268. doi: 10.1038/srep11268 (PMC4462756; doi:10.1038/srep11268)
Supplement: Supplementary Information [file srep11268-s1.pdf]

The p53 binding protein PDCD5 is not rate-limiting in DNA damage induced cell death

Florian J. Bock, Maria C. Tanzer, Manuel D. Haschka, Gerhard Krumschnabel, Bénédicte Sohm, Katrin Goetsch, Reinhard Kofler, and Andreas Villunger

a

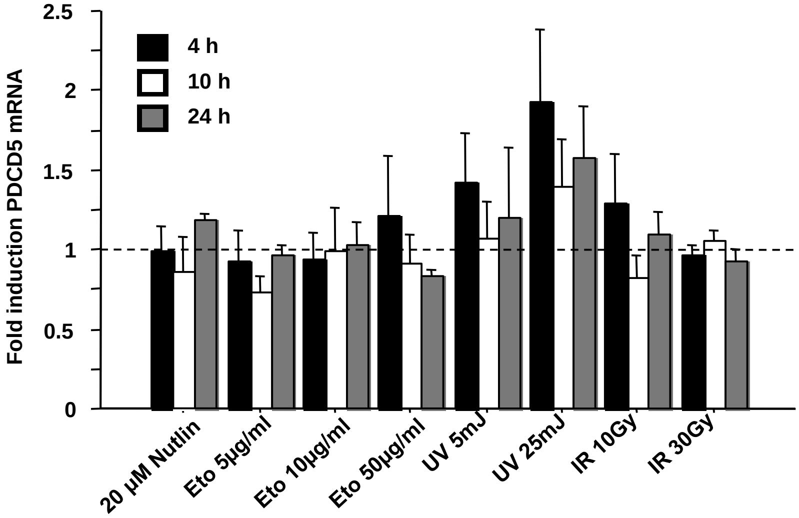

b

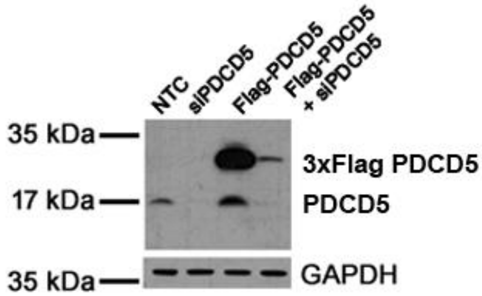

(a) Hct116 cells were treated as indicated for different times and PDCD5 mRNA levels relative to untreated cells were quantified by RT-qPCR. Data represent mean ± SEM; n ≥ 3.

(b) 293T cells were transfected with siPDCD5, Flag-tagged PDCD5 or both and immunoblotted for PDCD5 and GAPDH to determine specificity of the newly generated PDCD5 antiserum. NTC = non-transfected control.

Bock et al. Suppl. Figure 1

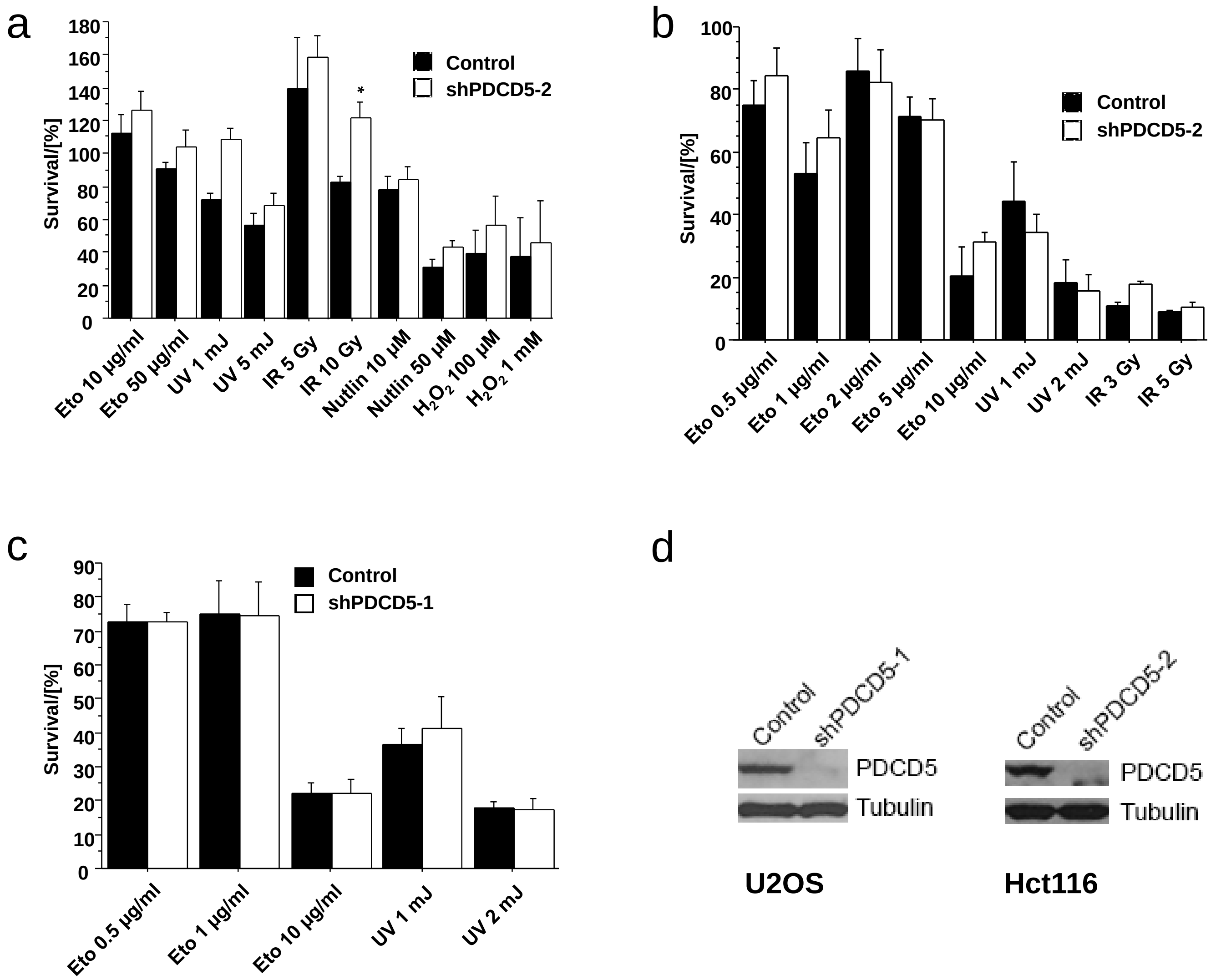

(a) Hct116 lines harbouring an independent inducible shRNA targeting PDCD5 were treated as indicated. After 24 h MTT assay was performed and survival determined relative to untreated cells. Data represent mean ± SEM; n = 3; *: $p<0.05$.

(b) The same Hct116 lines as in (a) were treated as indicated and colony formation was determined as specified in the material and methods section. Survival was calculated relative to untreated controls. Data represent mean ± SEM; n ≥ 3.

(c) U2OS lines harbouring an inducible shRNA targeting PDCD5 were treated as indicated and colony formation was determined as specified in the material and methods section. Survival was calculated relative to untreated controls. Mean ± SEM; n ≥ 3.

(d) Knockdown efficiency for shPDCD5-1 in U2OS cells and shPDCD5-2 in Hct116 cells was determined by western blotting.

Bock et al. Suppl. Figure 2

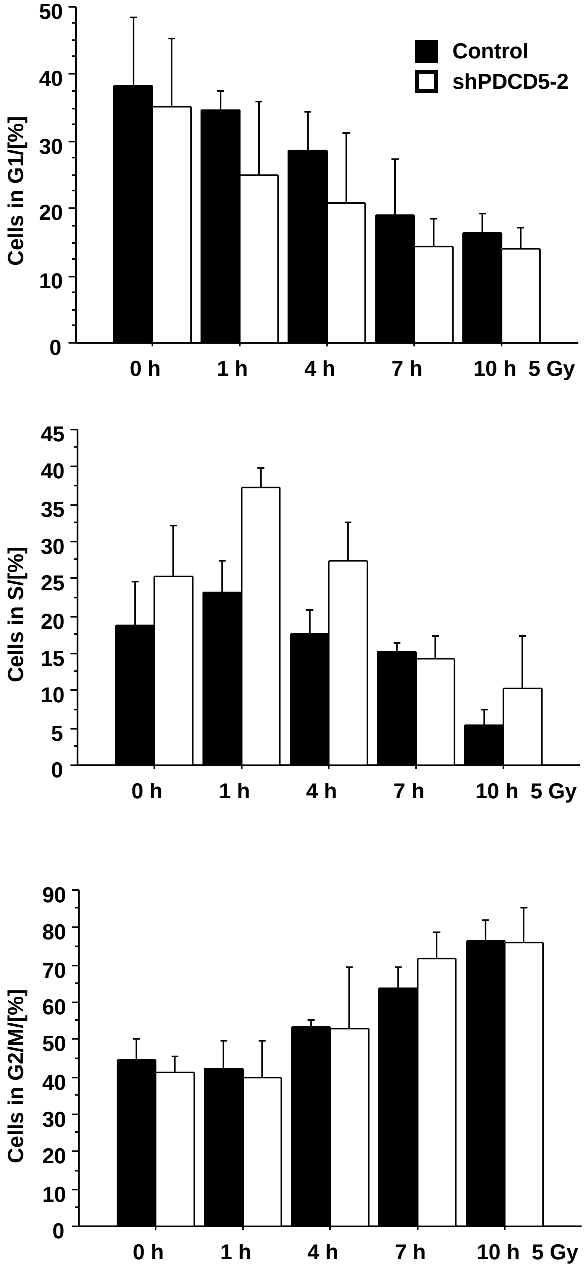

Hct116 cells with inducible PDCD5 shRNA were treated with doxycycline for 5 days followed by γ-irradiation for the indicated times. Cell cycle distribution was assessed by PI staining and quantified by flow cytometry. Data represent mean ± SD; n = 2
